# MIAT Is a Pro-fibrotic Long Non-coding RNA Governing Cardiac Fibrosis in Post-infarct Myocardium

**DOI:** 10.1038/srep42657

**Published:** 2017-02-15

**Authors:** Xuefeng Qu, Yue Du, You Shu, Ming Gao, Fei Sun, Shenjian Luo, Ti Yang, Linfeng Zhan, Yin Yuan, Wenfeng Chu, Zhenwei Pan, Zhiguo Wang, Baofeng Yang, Yanjie Lu

**Affiliations:** 1 Department of Pharmacology (State-Province Key Laboratories of Biomedicine-Pharmaceutics of China, Key Laboratory of Cardiovascular Research, Ministry of Education), College of Pharmacy, Harbin Medical University, Harbin, P.R China; 2 Northern Translational Medicine Research and Cooperation Center, Heilongjiang Academy of Medical Sciences, Harbin Medical University, Harbin, Heilongjiang 150081, P. R. China

## Abstract

A long non-coding RNA (lncRNA), named myocardial infarction associated transcript (MIAT), has been documented to confer risk of myocardial infarction (MI). The aim of this study is to elucidate the pathophysiological role of MIAT in regulation of cardiac fibrosis. In a mouse model of MI, we found that MIAT was remarkably up-regulated, which was accompanied by cardiac interstitial fibrosis. MIAT up-regulation in MI was accompanied by deregulation of some fibrosis-related regulators: down-regulation of miR-24 and up-regulation of Furin and TGF-β1. Most notably, knockdown of endogenous MIAT by its siRNA reduced cardiac fibrosis and improved cardiac function and restored the deregulated expression of the fibrosis-related regulators. In cardiac fibroblasts treated with serum or angiotensin II, similar up-regulation of MIAT and down-regulation of miR-24 were consistently observed. These changes promoted fibroblasts proliferation and collagen accumulation, whereas knockdown of MIAT by siRNA or overexpression of miR-24 with its mimic abrogated the fibrogenesis. Our study therefore has identified MIAT as the first pro-fibrotic lncRNA in heart and unraveled the role of MIAT in the pathogenesis of MI. These findings also promise that normalization of MIAT level may prove to be a therapeutic option for the treatment of MI-induced cardiac fibrosis and the associated cardiac dysfunction.

Myocardial infarction (MI) is the leading cause of death in the worldwide^1^,^2^, which is characterized by myocardial remodeling processes involving left ventricular (LV) dilation, cardiomyocyte hypertrophy, arrhythmias, cardiac fibrosis, and cell death accompanied by altered expression of genes, leading to heart failure (HF)^3^,^4^,^5^. A large plethora of cellular factors, including functional proteins and regulatory RNAs, are contributing to the remodeling processes as manifested by functional anomaly of these factors and/or expression deregulation of the genes encoding these factors^6^,^7^,^8^,^9^,^10^,^11^. The factors involved are intricate and remain incompletely understood. Following the recent discovery of microRNAs (miRNAs) for their indispensable roles in regulating expression of protein-coding genes, another class of functional RNAs called long non-coding RNAs (lncRNAs) has more recently emerging as key regulators of gene expression and the cellular functions these genes undertake. LncRNAs are mRNA-like transcripts ranging 200 to 100 kb nucleotides in length lacking protein-coding activity, yet are participating in many fundamental biological processes and pathophysiological events. In addition to their relatively well-described roles in human cancers and neuronal diseases, lncRNAs have also begun to be recognized for their functions in cardiovascular disease^12^,^13^,^14^,^15^,^16^,^17^. A finding particularly relevant to the present work is the discovery documented by Ishii *et al*. that myocardial infarction associated transcript (MIAT), a lncRNA was identified through a large-scale case-control association study confers risk of MI due to its altered expression by the single nucleotide polymorphism and has a significant association with the pathogenesis of MI^18^. Whilst this study unraveled an epigenetic factor that controls MI representing a major research breakthrough in the field of lncRNA and cardiovascular disease and an advancement of our understanding of lncRNA functions, what precise role that MIAT plays in the pathogenesis of MI and how exactly MIAT takes its actions have not been experimentally investigated. Thus, the exact cellular function of MIAT and the associated molecular mechanisms remain unknown.

Pathological changes after MI are a long-term process and cardiac fibrosis is an inevitable event in this process, which brings about an initial benefit and ultimate damage aftermath. On one hand, generation and maintenance of the extracellular matrix (ECM) in the scar is essential for preventing dilatation of the infarct zone leading to HF. On the other hand, ECM deposition at regions remote from the infarct area can result in excessive collagen production thereby cardiac stiffness that constitutes the basis of the development of HF. Because of this delicate balance between good and bad, delicate regulation of cardiac fibrosis is absolutely required by highly evolved creatures like our human beings. Indeed, cardiac fibrosis is an intricate process orchestrated by a wide spectrum of causal factors and regulatory factors and their mediated signaling pathways. Thorough understanding of the regulation of cardiac fibrosis is therefore imminently desirable for more rational management of MI and MI-induced development of HF associated with fibrosis.

Among various fibrosis-regulating pathways, the transforming growth factor β1 (TGF-β1) signaling pathway is of paramount importance^7^,^8^. It is known that while TGF-β1 released at an early stage of MI act as cardioprotective factor, presumably via the non-canonical pathway, TGF-β1 released at later time points post MI can lead to apoptosis, hypertrophy, and fibrosis. In addition to well-characterized functional regulation of TGF-β1 activation, TGF-β1 expression is also under delicate regulation at both transcriptional and post-transcriptional levels. Our pilot study predicted that MIAT has the potential to act as a competing endogenous RNA (ceRNA) to regulate TGF-β1 expression by competing for a shared miRNA miR-24, as the former contains a sequence domain highly complementary to the latter that has been reported to be a regulator of TGF-β1 activation^11^. This initial finding formed the basis of the present study and urged us to perform a series of experiments to elucidate the pathophysiological role of MIAT in MI and regulation of cardiac fibrosis as a potential mechanism.

## Results

### Abnormal Upregulation of MIAT in Infarcted Myocardium

**Figure 1:**
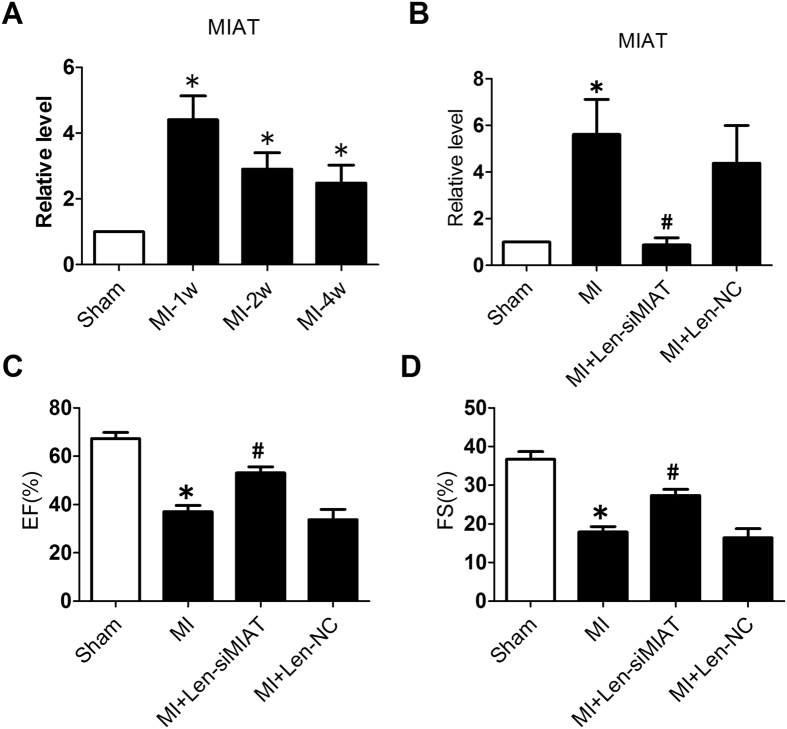
Expression deregulation of a long non-coding RNA myocardial infarction associated transcript (MIAT) and its role in cardiac dysfunction in a mouse model of myocardial infarction (MI). (**A**) Up-regulation of MIAT in the peri-infarct zone of MI hearts (n = 8/each group). (**B**) Level of MIAT after infarction and Len-siMIAT treatment. *P < 0.05 vs sham; ^#^P < 0.05 vs MI; n = 4. (**C**) and (**D**) Ejection fraction (EF%) and fractional shortening (FS%) examined by echocardiography. *P < 0.05 *vs* sham-operation control mice; ^#^*P* < 0.05 *vs* MI alone; n = 9 to 11/each group mice.

Myocardial infarction associated transcript (MIAT) has been reported to be associated with the pathogenesis of myocardial infarction (MI)^18^. In the present study, expression level of MIAT was robustly increased in the peri-infarct region at 1, 2, and 4 weeks following MI. The changes of MIAT expression was bi-phasic with an initial increasing phase peaking at 1 week followed by a subsequent descending phase thereafter (Fig. 1A). MIAT level in MI hearts remained as high as 2.5 times that in the hearts from the sham-operated control animals.

### Knockdown of MIAT Improves Cardiac Function in Infarcted Heart

To investigate if MIAT up-regulation in MI can produce any consequences on the pathological process or is merely a consequence of myocardial ischemia, we assessed the effects of MIAT knockdown on cardiac function in the context of MI. We first injected the lentiviral vectors carrying siMIAT (Len-siMIAT) into left ventricular chamber of mice and then occluded the left descending coronary artery (LAD). We then performed echocardiographic measurements 4 weeks after siMIAT administration. The aberrant upregulation of MIAT in MI hearts was significantly inhibited by the administration of Len-siMIAT (lentivirus vector carrying siRNA-MIAT, Fig. 1B). As summarized in Table 1, the MI hearts were significantly dilated, as reflected by the increases in left ventricular internal diastolic diameter (LVIDd) and left ventricular internal systolic diameter (LVIDs), and by the decreases in interventricular septum diastolic thickness (IVSDT) and interventricular septum systolic thickness (IVSST). These structural/morphological deteriorations substantially impaired cardiac function, as indicated by markedly reduced fractional shortening (EF) and ejection fraction (FS) (Fig. 1C,D). Len-siMIAT significantly attenuated these deleterious changes induced by MI; by comparison, the lentivirus carrying an empty vector (Len-NC) failed to affect the anomalies (Fig. 1C,D; Table 1).

### Knockdown of MIAT Inhibits Interstitial Fibrosis by Inhibiting Collagen Production and Cardiac Fibroblast (CF) Proliferation

**Figure 2:**
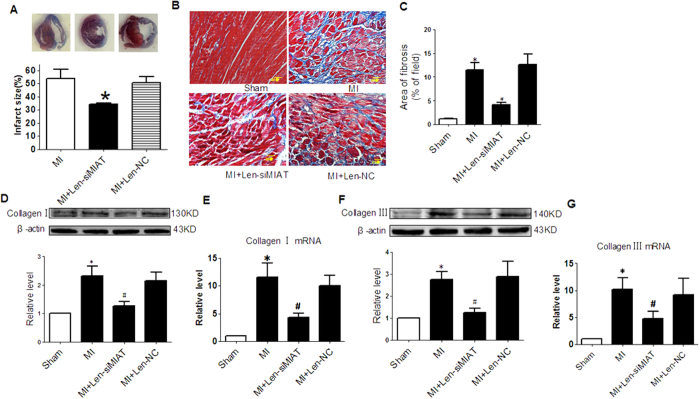
Role of MIAT in cardiac fibrosis of infarct hearts. (**A**) Infarct size of MI hearts after Len-siMIAT treatment. *P < 0.05 MI, n = 4. (**B**) Representative images of Masson trichrome staining with interstitial fibrosis in blue. (**C**) Mean values of the ratio of collagen surface area to the myocardial surface area expressed as percentage of fibrosis. *P < 0.05 *vs* sham-operation control hearts; ^#^*P* < 0.01 *vs* MI; n = 4 hearts. (**D**) and (**E**) Proetin and mRNA levels of collagen I after silence of MIAT by siRNA. *P < 0.05 *vs* sham; ^#^*P* < 0.05 *vs* MI; n = 5. (**F**) and (**G**) Proetin and mRNA levels of collagen III after silence of MIAT by siRNA. *P < 0.05 *vs* sham; ^#^*P* < 0.05 *vs* MI; n = 5.

These above results clearly indicated that MIAT up-regulation observed in our model was significantly involved in the dysfunction of MI hearts. This notion encouraged us to further exploit the role of MIAT in the heart. We chose to study interstitial fibrosis as it is a major factor leading to increased myocardial stiffness and depressed cardiac compliance after MI and also our preliminary analysis has suggested the potential role of MIAT in regulating fibrosis. To this end, we measured the infarct size and ECM deposition in the peri-infarct region with Masson trichrome staining. The infarct size was markedly reduced in Len-siMIAT treated mice in comparison with the control mice 1 week after MI (Fig. 2A). The interstitial fibrotic area of MI mice was significantly increased compared with sham controls, and transfection of Len-siMIAT significantly attenuated ECM deposition (Fig. 2B and C). Len-NC had no effect on fibrosis. Moreover, alleviation of post infarct ECM deposition by Len-siMIAT was also confirmed by decreased collagen I and collagen III levels in peri-infarct tissue (Fig. 2D–G).

**Figure 3:**
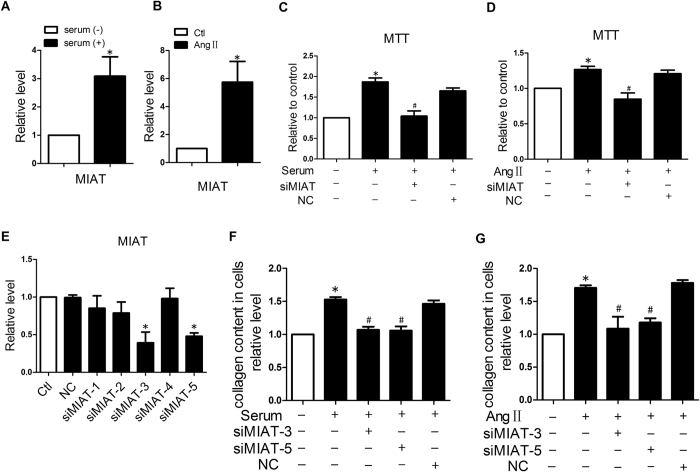
Effect of MIAT knockdown on the proliferation and collagen production of cultured cardiac fibroblasts. (**A**) and (**B**) Up-regulation of MIAT expression in cultured cardiac fibroblasts treated with high serum or angiotensin II (AngII) to simulate fibrogenesis, resembling that observed in MI hearts. *P < 0.05 *vs* mock-treated control; n = 5 batches of cells. (**C**) and (**D**) Effects of MIAT on proliferation of cardiac fibroblasts determined by MTT assay. Note that serum (left) or AngII (right) stimulated proliferation of cardiac fibroblasts, but the growth was suppressed by siMIAT to knockdown endogenous MIAT. *P < 0.05 *vs* mock-treated negative control; ^#^*P* < 0.05 *vs* Serum or AngII alone; n = 4 to 5 batches of cells. (**E**) Verification of the efficiency of 5 siMIATs in knocking-down endogenous MIAT in cultured cardiac fibroblasts. (**F**) and (**G**) Effects of siMIAT-3 and siMIAT-5 on the collagen contents induced by serum or AngII. *P < 0.05 *vs* mock-treated negative control; ^#^*P* < 0.05 *vs* Serum or AngII alone; n = 4 batches of cells. *P < 0.05 *vs* mock-treated negative control. n = 4 batches of cells.

Next, we wanted to know whether the MI-induced up-regulation of MIAT takes place in cardiac fibroblasts. We first treated the cultured NMCFs with 20% serum or 150 nM angiotensin II (AngII) for 24 h, and found that MIAT expression was up-regulated by 3.1 ± 0.7 and 5.8 ± 1.5 times respectively compared with control group (Fig. 3A and B). In addition, 20% serum or AngII remarkably boosted up NMCF proliferation, whereas siMIAT abrogated these changes (Fig. 3C and D). We then detected total collagen production using a colorimetric reaction with picrosirius red in cultured NMCFs. AngII or serum treatment considerably increased the total collagen contents compared with the mock-treated control cells. As expected, siMIAT abolished these pro-fibrotic alterations (Fig. 3F and G). The scrambled negative control siRNA (siNC) had no effects on AngII- or serum-induced NMCF collagen production.

The efficacy of siMIAT to knockdown endogenous MIAT was examined in the NMCFs. Of the 5 siRNA for MIAT, 2 siRNAs (siMIAT-3 and siMIAT-5) reduced the expression of MIAT by approximately 60% and 52%, while siMIAT-1, siMIAT-2, and siMIAT-4 did not produce such effects in NMCFs. siNC (negative control siRNA) showed no appreciable effects on MIAT levels (Fig. 3E). As expected, siMIAT-3 and siMIAT-5 exhibited significant inhibitory effects on AngII or serum-induced collagen production. siNC did not show influence on collagen production (Fig. 3F and 3G).

### MIAT Functions as MiR-24 Sponge in CFs

**Figure 4:**
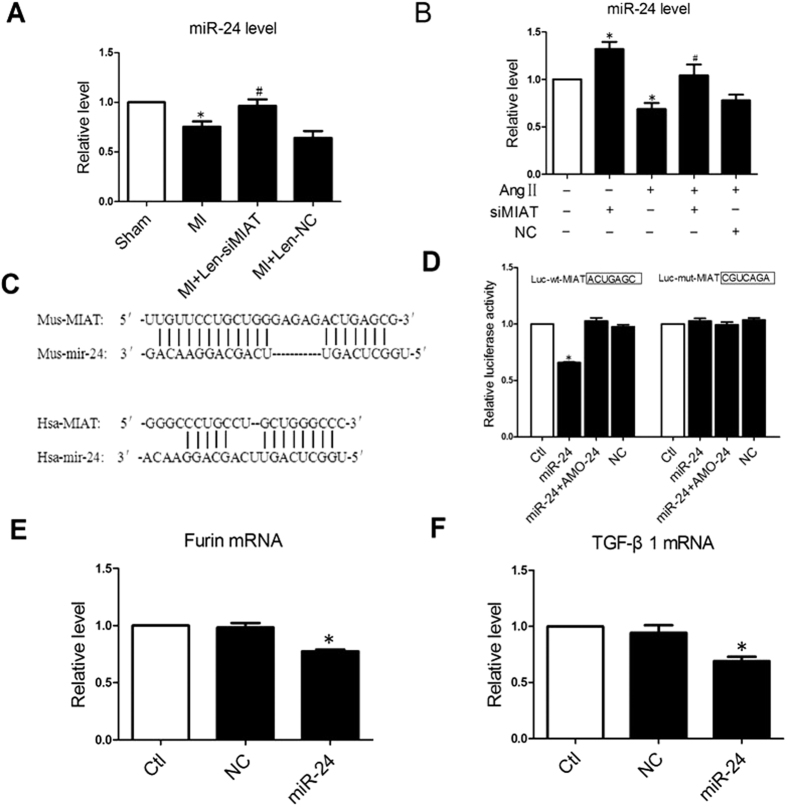
Regulation of miR-24 level by MIAT. (**A**) Down-regulation of miR-24 and rescuing by Len-siMIAT to knockdown endogenous MIAT in MI hearts. *P < 0.05 *vs* sham-operation control hearts; ^#^*P* < 0.05 *vs* MI alone; n = 5 hearts. (**B**) Down-regulation of miR-24 and rescuing by siMIAT in cultured cardiac fibroblasts treated by angiotensin II (AngII) to stimulate proliferation. *P < 0.05 *vs* mock-treated control and ^#^*P* < 0.05 *vs* AngII alone; n = 3 to 4 batches of cells. (**C**) Sequence alignment showing the complementarity between MIAT and miR-24. “|” indicates base pairing. (**D**) Luciferase reporter gene assay showing the direct functional interactions between MIAT and miR-24 in HEK293 cells. Note that transfection of miR-24 mimic significantly reduced the luciferase activities elicited by the vector containing a fragment of MIAT sequence spanning the miR-24 binding site (Luc-wt-MIAT), an effect reversed by the miR-24 antisense inhibitor AMO-24. Mutation of the binding site in the fragment (Luc-mut-MIAT) rendered a failure of miR-24 to affect luciferase activities. *P < 0.05 *vs* mock-treated control; n = 3 experiments. (**E** and **F**). Furin and TGF-β1 mRNAs level in CFs transfected with miR-24 mimics. n = 4 experiments. *P* < 0.05 *vs* NC (negative control).

Recent studies have demonstrated that lncRNAs can act as competing endogenous RNAs (ceRNAs) or endogenous sponge RNAs to bind miRNAs by sequence complementarity so as to absorb them and decrease the availability of the functional miRNAs in the cell^16^,^19^. In the present study, we consistently observed remarkable decreases in miR-24 level in the border region of the MI hearts and this down-regulation was reversed in mice treated with Len-siMIAT (Fig. 4A). In NMCFs, miR-24 expression was substantially mitigated after AngII treatment but was up-regulated when MIAT had been knocked down by siMIAT (Fig. 4B). These reciprocal changes in expression in myocardium and NMCFs between MIAT and miR-24 suggested a possible interactive relationship. In accordance with this notion, computational analysis revealed significant sequence complementarity between miR-24 and MIAT (Fig. 4C). More intriguingly, miR-24 has been previously characterized with its regulatory role in ischemia-induced cardiac fibrosis through targeting Furin, a component of the TGF-β1 signaling pathway^11^. We therefore focused our subsequent investigation on miR-24.

We used luciferase reporter gene assay to establish the direct functional interactions between MIAT and miR-24. As illustrated in Fig. 4D, co-transfection of the luciferase vector carrying the wild-type MIAT fragment (Luc-wt-MIAT) with miR-24 mimic into HEK293 cells caused an enormous diminishment of luciferase activities relative to Luc-wt-MIAT alone. This effect of miR-24 was not seen when the MIAT fragment was mutated (Luc-mut-MIAT) to destroy the binding sequence for miR-24. Moreover, we confirmed the regulation of miR-24 on Furin and TGF-β1 by the finding that overexpression of miR-24 in CFs inhibited the mRNA expression of both Furin and TGF-β1 (Fig. 4E,F)

**Figure 5:**
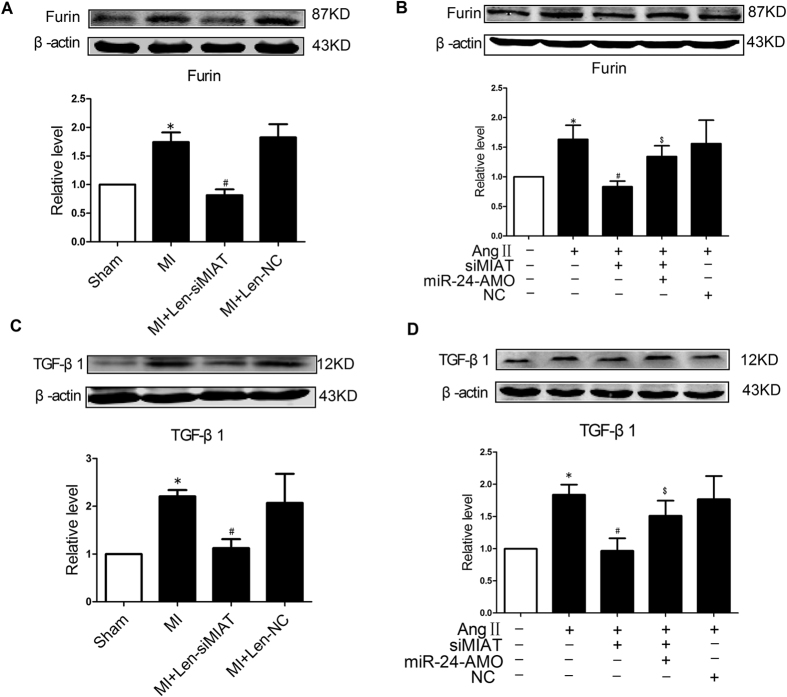
Effects of MIAT on the protein levels of Furin and TGF-β1. (**A**) Len-siMIAT to knockdown endogenous MIAT abrogated MI-induced up-regulation of Furin in a mouse model of MI. *P < 0.05 *vs* sham-operation control hearts; ^#^*P* < 0.05 *vs* MI alone; n = 5 hearts. (**B**) Silence of MIAT by siMIAT reversed AngII-induced up-regulation of Furin in cultured cardiac fibroblasts. Note that knockdown of miR-24 by AMO-24 partially prevented the effects of siMIAT on Furin expression. *P < 0.05 *vs* mock-treated control, ^#^*P* < 0.05 *vs* AngII alone, and ^$^*P* < 0.05 *vs* siMIAT in the presence of AngII; n = 4 batches of cells. (**C**) Len-siMIAT to knockdown endogenous MIAT abrogated MI-induced up-regulation of TGF-β1 in a mouse model of MI. *P < 0.05 *vs* sham-operation control hearts, and ^#^*P* < 0.05 *vs* MI alone; n = 6 hearts. (**D**) Silence of MIAT by siMIAT reversed AngII-induced up-regulation of TGF-β1 in cultured cardiac fibroblasts. Note that knockdown of miR-24 by AMO-24 partially prevented the effects of siMIAT on Furin expression. *P < 0.05 *vs* mock-treated control, ^#^*P* < 0.05 *vs* AngII alone, and ^$^*P* < 0.05 *vs* siMIAT in the presence of AngII; n = 5 batches of cells.

The above results strongly suggested that up-regulation of MIAT in the setting of MI could dramatically reduce the availability of functional miR-24 in the heart, or more specifically in CFs. This thought prompted us to continue our investigation on the changes of expression of Furin, a target gene for miR-24. Our data demonstrated that Furin was significantly up-regulated in its expression at the protein level in peri-infarct region of the MI hearts (Fig. 5A). In sharp contrast, Furin was found down-regulated in the mice pretreated with Len-siMIAT, but unaffected by Len-siNC. Similarly, Furin was up-regulated after AngII treatment in NMCFs (Fig. 5B), being inversely related to the changes of miR-24 expression (Fig. 4B); but in cells transfected with siMIAT Furin level became reduced (Fig. 5B).

Furin is a proprotein convertase enzyme that process latent precursor proteins into their biologically active products and it plays an important role in TGF-β1 activation (increase in functional TGF-β1 protein level) leading to cardiac fibrogenesis^11^. We therefore moved further to monitor the changes of the protein levels of TGF-β1 under varying conditions. TGF-β1 protein level was substantially higher in the MI hearts compared to the sham control (Fig. 5C), but this difference was eliminated by Len-siMIAT. Similarly at the cellular level in NMCFs, AngII elevated TGF-β1 level but knockdown of MIAT by siMIAT attenuated the stimulating effect of AngII on TGF-β1 (Fig. 5D). Furthermore, miR-24 antisense inhibitor (AMO-24) effectively prevented the siMIAT-induced decreases in Furin and TGF-β1 protein levels (Fig. 5B and D).

## Discussion

LncRNAs have been increasingly recognized for their roles in shaping cardiac structure and function and defining the pathogenesis of cardiovascular disease. Like miRNAs, these regulatory molecules are often able to cause well-defined phenotypical changes of cells, tissues, organs or even the whole organisms. A number of lncRNAs have recently been identified for their roles in cardiovascular pathology^17^,^19^,^20^, among which MIAT stands out as a particularly attractive one as it confers risk of myocardial infarction (MI) by a genetic variant^18^ and may serve as a biomarker for MI, in addition to regulating microvascular function in the setting of diabetes^21^. Nonetheless, despite its apparent significance in MI, MIAT has not been experimentally studied for its specific role and the mechanisms of action. From the broader view, despite an order of magnitude more prevalent than miRNAs, lncRNAs as a new layer of gene expression regulatory network have been scarcely exploited for their cellular functions and pathological roles in the heart, partly due to the technical difficulties in studying the mechanisms of actions of these molecules. The present study made a step forward to improving our understanding of lncRNAs by focusing on MIAT in the setting of cardiac fibrosis induced by MI with an *in vivo* model and by AngII in an *in vitro* cellular model with cardiac fibroblasts.

Though a number of lncRNAs have been linked to cardiovascular disease through genetic linkage studies^18^,^22^,^23^,^24^, detailed experimental research into the mechanistic links in animal or cellular models has been thus far scanty. The first experimental report by Wang *et al*.^17^ identified a lncRNA (designated cardiac hypertrophy related factor, CHRF) as a critical regulator of cardiac hypertrophy in both *in vivo* and *in vitro* models, which by working together with miR-489 and Myd88 constitutes a novel cardiac hypertrophy regulating system. Subsequently, the same group reported identification of another lncRNA named cardiac apoptosis-related lncRNA (CARL) that was shown to inhibit anoxia-induced mitochondrial fission and apoptosis in cardiomyocytes by relieving miR-539-dependent PHB2 repression^19^. A more recent study by Yan *et al*.^21^ demonstrated MIAT is involved in diabetes mellitus-induced retinal microvascular dysfunction by inhibiting endothelial cell proliferation, migration, and tube formation. To the best of our knowledge, there have not been any experimental studies on lncRNAs and the pathogenesis of MI. In particular, despite the recognized role of MIAT in rendering susceptibility to MI, how exactly MIAT contributes to the pathogenesis of MI has not been studied. Here, we provided the first evidence that MIAT is a pro-fibrotic factor that controls cardiac fibrosis and regulates cardiac function in the setting of MI. MIAT was enormously up-regulated in MI hearts and in CFs treated with AngII. This up-regulation contributed significantly to the post-infarct remodeling processes with impaired cardiac compliance and increased risk of HF as indicated by the deleterious alterations of the echocardiographic parameters. These are reflected by the electrifying restoration of normal cardiac structure (reduced fibrotic tissues) and function (improved echocardiographic parameters) upon normalization of MIAT level in the myocardium.

The mechanisms by which lncRNAs regulate gene expression are diverse and largely unpredictable based on their sequence information, except for their interactions with miRNAs. LncRNAs often contains sequence domains complementary to miRNAs, which confers their ability to bind miRNAs and serve as sponge of these small molecules so as to limit the availability of the targeted miRNAs^16^,^17^,^19^,^25^. In this way, lncRNAs indirectly regulate expression of specific protein-coding genes that are targets of the targeted miRNAs. Specifically, a lncRNA, by absorbing a target miRNA, can predictably up-regulate the target genes of that miRNA by relieving the repressive effects of the miRNA on the target gene. The competing endogenous RNA (ceRNA) hypothesis was proposed based on this mechanism of action. Today, ceRNA has become a popular research approach for discovering the cellular functions and the associated biological outcomes of lncRNAs. Indeed, CHRF, CARL, and MIAT have all been classified as a ceRNA because of their ability to bind endogenous miRNAs. For instance, lncRNA-CHRF regulates cardiac hypertrophy by competitively binding miR-489 that regulates Myd88 expression^17^. CARL can suppress mitochondrial fission and apoptosis by acting as an endogenous miR-539 sponge that regulates PHB2 expression^19^. And linc-MD1 can sponge miR-133 and miR-135 to regulate the expression of MAML1 and MEF2C, two transcription factors that can activate muscle-specific gene expression^16^. MIAT has been shown to suck miR-150-5p to regulate VEGF expression^21^. In our studies, we showed that MIAT reduced the detectable miR-24 in the heart and CFs, whereas knockdown of endogenous MIAT increased miR-24 level. Correspondingly and expectedly, Furin, a target gene for miR-24, displayed the opposite changes to miR-24 in terms of their expression levels. Furin is known to be an activating factor of TGF-β1 that dictates cardiac fibrosis in various conditions^11^,^26^. Consistent with this notion, we found that MIAT and TGF-β1 went along together in their expression levels: both were elevated by MI or AngII treatment, but lowered upon knockdown of MIAT. Most importantly, these changes coincided with CF proliferation, collagen production and fibrogenesis. These data point to the existence of a new cardiac fibrosis-controlling system: MIAT↑ → miR-24↓ → Furin/TGF-β1↑ → cardiac fibrosis↑. Consistent with this miR-24 has been found be down-regulated^11^, whereas Furin and TGF-β1 up-regulated in the MI heart. Whilst miR-24 down-regulation is believed to cause cardiac fibrosis, how it is down-regulated remained unknown. The present study revealed a mechanism for ischemic down-regulation of miR-24: up-regulation of MIAT rendered down-regulation of miR-24.

MI can result in death of ischemic cardiac tissue followed by ECM deposition/cardiac fibrosis and dysfunction of myocardial contraction. The adverse structural and functional remodeling processes during MI can eventually lead to heart failure. Preventing pathological fibrogenesis therefore has been regarded as a promising strategy for alleviating the remodeling processes thereby HF. Our data showed that preventing MIAT up-regulation by knocking down its expression significantly lessened the MI-induced cardiac fibrosis and restored the impaired cardiac function. At the cellular level, MIAT knockdown by a means of gene silencing techniques significantly inhibited cardiac fibroblast proliferation and collagen accumulation induced by high serum or AngII. These findings provided a new strategy for the management of MI hearts through targeting MIAT to limit cardiac fibrogenesis so as to retard the development of HF. From a broader perspective, MIAT may be considered a therapeutic target for other cardiac conditions associated with fibrosis. This conception should spur us into more future studies with rigorous and vigorous experimentations on MIAT and other lncRNAs as well.

One of the limitations of the present study is the lack of artificial gain-of-function approaches to experimentally address the adverse effects of MIAT. This is primarily due to the technical difficulty of manipulating a large size RNA like MIAT (8760 bp) for overexpression. Nevertheless, the fact that MIAT was “overexpressed” in MI hearts could be regarded as pathological gain-of-function. It should be noted that while our study focused on cardiac fibrosis in the context of MI, it does not exclude any other unknown cellular functions and pathological roles that MIAT can possibly elicit in the heart. Additionally, the mechanisms of MIAT function are likely multiplex and require more studies to elucidate.

With regard to the mechanisms by which MIAT takes its effects in cardiac fibrosis, our study focused on miR-24 only leaving other miRNAs (including such as miR-21^27^, miR-29^28^, miR-30, and miR-133^9^,^10^) unattended, which have been reported to contribute to cardiac fibrosis. We used RNAhybrid to predict interaction of MIAT and miRNAs and found that MIAT also contains binding sequence for miRNAs-29, 21, 133, 30, and 24. It has been reported that MIAT does not serve as a competing endogenous RNA for miR-29^21^. MiR-21 is considered a pro-fibrotic miRNA whose expression is upregulated in MI contributing to myocardial fibrosis^28^. The possibility of its direct interaction with MIAT can be excluded as they both are up-regulated in MI, indicating that miR-21 is not likely involved in the sponge mechanism of MIAT. Duisters *et al*. demonstrated that miR-133 and miR-30 participate in the process of cardiac fibrosis in hypertension-induced heart failure^9^. miR-133 also contributes to the development of cardiac fibrosis in diabetes^29^. Notably, miR-24 plays an important role in regulation of MI-induced cardiac fibrosis^11^ which was employed as an experimental model in our study. We therefore focused on miR-24 in the present study. On the other hand, miR-29, miR-30 and miR-133 are all anti-fibrotic miRNAs with their expression down-regulated to promote cardiac fibrosis in cardiac diseases, resembling miR-24. However, our study by no means rules out the possible participation of these miRNAs in MIAT-mediated cardiac fibrosis.

By demonstrating the critical involvement of MIAT in MI-induced cardiac fibrosis and dysfunction, our study unraveled the cellular function and pathological role of MIAT in the heart, suggesting lncRNAs as a new layer of the regulatory network governing cardiac fibrosis. The finding that MIAT can absorb miR-24 through its sponge-like action as a ceRNA provided an explanation for the ischemic down-regulation of miR-24 and allowed us to have establish a new fibrosis-regulatory modality MIAT↑ → miR-24↓ → Furin/TGF-β1↑ → cardiac fibrosis↑, which operates in MI and maybe in other cardiac pathological processes associated with fibrosis. Our study fills the gap between the cellular function of MIAT and its recognized property to confer risk of MI and should therefore advance our understanding of lncRNA functions in the heart. Meanwhile, as the first fibrosis-controlling lncRNA identified thus far, MIAT merits further detailed studies for its potential as a therapeutic target for suppressing cardiac fibrosis thereby impeding the development of heart failure.

## Methods

### Animals

Healthy male C57BL/6 mice (weighing 21–25 g, 12 to 16 weeks old) were provided by the Experimental Animal Center of the Harbin Medical University. Food and water were freely accessible by mice. All experimental procedures were performed in accordance with and approved by the Institutional Animal Care and Use Committee of the Harbin Medical University.

### Myocardial Infarction Model

Mice were anesthetized with Avertin (160 mg/kg, ip. Sigma-Alorich) and their chests were opened to expose the hearts. The left descending coronary artery (LAD) was ligated with a 7/0 nylon suture at 2 mm below the border between left atrium and ventricle to induce myocardial infarction. Myocardial ischemia was confirmed by significantly S-T segment elevation with electrocardiographic measurements. The sham-operated mice for control underwent the same experimental procedures as the MI group but without ligation of LAD.

After surgery mice were monitored daily for signs of infection and state of health and activity. The mice were anesthetized for echocardiography and hemodynamic measurements and sacrificed by cervical dislocation at 1, 2 and 4 weeks after MI.

### Construction of Lentivirus Carrying SiRNA of MIAT

MIAT siRNAs (siMIAT) and the negative control siRNA (siNC) were designed and purchased from Invitrogen (Shanghai, China). Their sequences are provided in Table 2. The constructs were inserted into lentivirus vector plasmid PHY-LV-KD5.1 (Invitrogen, Shanghai, China). A recombinant lentivirus vector carrying siRNA-MIAT was successfully constructed through homologous recombination method. The ascending aortic artery was clamped for tansfection of lentivirus plasmid. The plamid 70 μl (10^8^ TU/ml) carrying siMIAT or siNC was injected into the left ventricular chamber and was forced into the coronary arteries when aortic artery was temporarily occluded for 15 s. Sham group mice received DMEM 70 μl for control. After 15 min of injection, myocardial infarction was induced in the transfected mice.

### Echocardiographic Measurements

Four weeks after injection of lentivirus, transthoracic echocardiography was performed to monitor changes of the left ventricular function using ultrasound machine Vevo2100 high-resolution imaging system (VisualSonics, Toronto, ON, Canada) equipped with a 10-MH2 phased-array transducer with the M-mode recordings. Ventricular parameters were measured and analyzed including left ventricular internal diastolic diameter (LVIDd), left ventricular internal systolic diameter (LVIDs), interventricular septum diastolic thickness (IVSDT) and interventricular septum systolic thickness (IVSST), left ventricular ejection fraction (EF), and fractional shortening (FS). The mice were killed with overdose sodium pentobarbital after measurements and the hearts were quickly removed and placed in liquid nitrogen or 4% paraformaldehyde for later uses.

### Masson Trichrome Staining

Mice were anesthetized using Avertin (160 mg/kg, ip. Sigma-Alorich) and the hearts were collected and fixed in 4% paraformalin for 24 h, embedded with paraffin, then cut into 5 μm thick cross-sectional slices along the center of the fibrotic scar. The slices were stained by Masson trichrome. Fibrotic tissue was stained blue and myocardium stained red. The fibrotic areas are calculated with image analysis software (Image-Pro plus v4.0; Meida Cybernetics, Bethseda, MD, USA). We used the percentage of fibrotic tissue area (blue area) to the myocardial surface area (red area) and fibrotic area (blue area) to obtain the collagen volume fraction for evaluation of the cardiac fibrosis.

### Primary Culture and Drug Treatment of Neonatal Mouse Cardiac Fibroblasts (NMCFs)

NMCFs were isolated as described previously in detail^6^. In brief, NMCFs were isolated from 2-day-old mice with 0.25% trypsin and cultured in DMEM supplemented with 10% fetal bovine serum (FBS). At 80% confluence they were treated with FBS (20%) or AngII (150 nM) after free serum for 24 h, followed by transfection with siMIAT (100 nM) or other constructs at same concentration for 48 h. X-tremeGene siRNA transfection reagent (Roche, USA) was used for the transfection of siRNA into cells.

### Cell Viability Assay

Cell viability was assessed by measuring the mitochondrial dependent reduction of 3-(4,5-dimethylthiazol-2-yl)-2,5-diphenyltetrazolium bromide (MTT), as previously described^6^. Cells (1 × 10^4^) were seeded on a 96-well plates and treated as designated. At 80% confluence, 15 μl MTT solution (Sigma-Aldrich, St. Louis, MO) was added into each well and the plates were incubated for 4 h. Then 200 μl of DMSO was added into each well to dissolve the formazan. The optical density was measured at 490 nm using a microplate reader (Tecan, Austria).

### Western Blotting

Total protein samples were extracted from NMCFs or left ventricular peri-infarct region (1–2 mm area between the infarct region and normal tissue) and corresponding regions of sham-operated hearts were collected 1, 2, 4 weeks after MI. Immunoblotting analysis was performed as already described elsewhere^6^. In brief, the total protein was electrophoresed on SDS-PAGE (15% poly-acrylamide gels) and blotted to nitrocellulose membrane. The primary antibodies were used as follows: anti-TGF-β1 (1:500, rabbit polyclonal, Abcom, USA), anti-Furin (1:500, rabbit polyclonal, Abcom, USA). The Western blot bands were captured by Imaging System (LI-COR Biosciences, Lincoln, NE, USA) and analyzed with odyssey v1.2 software.

### Quantitative Reverse Transcription-PCR (qRT-PCR)

Total RNA samples were extracted from cultured neonatal mouse CFs or left ventricular infarct border zone of heart using Trizol reagent (Invitrogen, USA). For each sample, 0.5 μg of the total RNA was converted to cDNA using high-capacity cDNA reverse transcription kit (Applied Biosystems, USA). RNA levels of MIAT, miR-24, collagen I (Col I) and collagen III (Col III) were detected by SYBR Green method on the ABI 7500 fast real-time PCR system (Applied Biosystems, USA). After circle reaction, the threshold cycle (Ct) was determined and the relative quantitative expression of MIAT, miR-24, and mRNAs was calculated using method 2^−^^Ct^ and normalized to β-actin as an internal control. The sequences of primers were synthesized by Guangzhou Ribo Biotechnology Co. Ltd (Guangzhou, China) and are listed in Table 3.

### Luciferase Assays

A fragment of the mouse MIAT gene containing the miR-24 binding site (designated Luc-wt-MIAT) was PCR amplified, and so was a fragment with nucleotide replacement mutation (designated Luc-mut-MIAT). For luciferase reporter gene assay, HEK293 cells were cultured in 24-well culture plates and were transfected with varying constructs as to be specified below. Firefly and Renilla luciferase activities were measured using the Dual Luciferase Reporter Assay System (Promega, Madison, WI, USA).

### Measurement of Collagen Content

NMCFs after a variety of treatments were lysed in 0.05 M Tris buffer (pH 7.5), and the total collagen was extracted with picrosirius red in saturated picric acid. Collagen contents were measured using essentially the same procedures as previously reported^30^, and were calculated based on the linear calibration curve constructed from standards and normalized to the total protein content.

### Statistics

Data are expressed as mean ± SEM in the present study. Statistical analyses were performed using one-way ANOVA and Student’s test. A two-tailed *P* < 0.05 was taken to indicate a statistically significant difference.

## Additional Information

**How to cite this article:** Qu, X. *et al*. MIAT Is a Pro-fibrotic Long Non-coding RNA Governing Cardiac Fibrosis in Post-infarct Myocardium. *Sci. Rep.* **7**, 42657; doi: 10.1038/srep42657 (2017).

**Publisher's note:** Springer Nature remains neutral with regard to jurisdictional claims in published maps and institutional affiliations.

## Figures and Tables

**Table 1:** HW/BW coefficient and cardiac function of MI mice treated with siMIAT for 4 weeks.

	Sham (n = 10)	MI (n = 11)	MI + SI (n = 10)	MI + NC (n = 9)
HW, mg	126.99 ± 1.78	188.16 ± 10.12*	171.81 ± 7.02	218.4 ± 7.71
BW, g	23.05 ± 0.45	25.49 ± 0.71	25.73 ± 0.42	27.13 ± 0.7
HW/BW, mg/g	5.64 ± 0.08	7.45 ± 0.31*	6.68 ± 0.25	8.06 ± 0.24
HR (BPM)	407.13 ± 15.98	433.91 ± 13.31	406.22 ± 14.96	423.89 ± 12.6
IVSDT, mm	0.80 ± 0.02	0.62 ± 0.08*	0.92 ± 0.05#	0.72 ± 0.09
IVSST, mm	1.24 ± 0.06	0.86 ± 0.12*	1.37 ± 0.07#	0.99 ± 0.13
LVIDd, mm	3.33 ± 0.07	4.56 ± 0.09*	4.40 ± 0.18	5.07 ± 0.21
LVIDs, mm	2.11 ± 0.07	3.74 ± 0.11*	3.19 ± 0.14#	4.24 ± 0.22
EF, %	67.31 ± 2.4	37.05 ± 2.46*	53.15 ± 2.37#	33.75 ± 3.83
FS, %	36.74 ± 1.87	17.91 ± 1.33*	27.36 ± 1.48#	16.43 ± 2.09

MI indicates myocardial infarction; SI, lentivirus carrying siRNA-MIAT; NC, empty lentivirus; HW, heart weight; BW, body weight; HW/BW, ratio of HW to BW; HR, heart rate; LVIDd, left ventricular diastolic diameter; LVIDs, left ventricular systolic diameter; IVSDT, interventricular septum diastolic thickness; IVSST, interventricular septum systolic thickness; FS, fractional shortening; and EF, ejection fraction. Data are expressed as mean ± SEM, ^*^P < 0.05 *vs* sham; ^#^*P* < 0.05 *vs* MI.

**Table 2:** Sequences of siRNA for MIAT.

RNA Name	Sequence(from 5′ to 3′ ends)
siMIAT-1 sense	CCUUACCAUUCCUCCACUUTT
siMIAT-1 anti-sense	AAGUGGAGGAAUGGUAAGGTT
siMIAT-2 sense	GCUGGCCACAUUAUAGAAATT
siMIAT-2 anti-sense	UUUCUAUAAUGUGGCCAGCTT
siMIAT-3 sense	CCAGGCUCCUUUAAACCAATT
siMIAT-3 anti-sense	UUGGUUUAAAGGAGCCUGGTT
siMIAT-4 sense	GCUCCUUGUUCGGUUUAUATT
siMIAT-4 anti-sense	UAUAAACCGAACAAGGAGCTT
siMIAT-5 sense	GCAGUUCUUAGCUCAUAUATT
siMIAT-5 anti-sense	UAUAUGAGCUAAGAACUGCTT

**Table 3:** Sequences of primers used for real time PCR analysis.

RNA name	Primers
For mouse β-actin, forward primer:	5′-ACTGCCGCATCCTCTTCT-3′;
reverse primer:	5′-TCAACGTCACACTTCATGATGGA-3′.
For mouse MIAT, forward:	5′- TGGAACAAGTCACGCTCGATT-3′;
reverse:	5′-GGTATCCCAAGGAATGAAGTCTGT-3′.
For mouse collagen I, forward:	5′-CAATGGCACGGCTGTGTGCG-3′;
reverse:	5′-CACTCGCCCTCCCGTCTTTGG-3′.
For mouse collagenIII, forward:	5′-TGGCACAGCAGTCCAACGTA-3′;
reverse:	5′-AAGGACAGATCCTGAGTCACAGACA-3′.
For mouse miR-24, forward:	5′-GGGGTGGCTCAGTTCAGCA-3′
reverse:	5′-CAGTGCGTGTCGTGGAGTC-3′
